# A New Method of Diatomaceous Earth Fractionation—A Bio-Raw Material Source for Epoxy-Based Composites

**DOI:** 10.3390/ma14071663

**Published:** 2021-03-28

**Authors:** Marta Dobrosielska, Renata Dobrucka, Michał Gloc, Dariusz Brząkalski, Marcin Szymański, Krzysztof J. Kurzydłowski, Robert E. Przekop

**Affiliations:** 1 Faculty of Materials Science and Engineering, Warsaw University of Technology, ul. Wołoska 141, 02-507 Warsaw, Poland; Marta.Dobrosielska@pw.edu.pl (M.D.); michal.gloc@pw.edu.pl (M.G.); krzysztof.kurzydlowski@pw.edu.pl (K.J.K.); 2 Department of Non-Food Products Quality and Packaging Development, Institute of Quality Science, Poznań University of Economics and Business, al. Niepodległości 10, 61-875 Poznań, Poland; 3 Faculty of Chemistry, Adam Mickiewicz University in Poznań, 8 Uniwersytetu Poznańskiego, 61-614 Poznań, Poland; d.brzakalski@gmail.com; 4 Centre for Advanced Technologies, Adam Mickiewicz University in Poznań, ul. Uniwersytetu Poznańskiego 10, 61-614 Poznan, Poland; marcin.szymanski@amu.edu.pl

**Keywords:** diatomite, bio-composites, mechanical properties, fractionation, purification of diatomaceous earth, bio-raw materials

## Abstract

The authors of this paper use an original method of diatomaceous earth fractionation, which allows for obtaining a filler with a specific particle size distribution. The method makes it possible to separate small, disintegrated and broken diatom frustules from those which maintained their original form in diatomaceous earth. The study covers a range of tests conducted to prove that such a separated diatomic fraction (3–30 µm) shows features different from the base diatomite (from 1 to above 40 µm) used as an epoxy resin filler. We have examined the mechanical properties of a series of diatomite/resin composites, considering the weight fraction of diatoms and the parameters of the composite production process. The studied composites of Epidian 601 epoxy resin cross-linked with amine-based curing agent Z-1 contained 0 to 70% vol. of diatoms or diatomaceous earth. Samples were produced by being casted into silicone molds in vacuum degassing conditions and, alternatively, without degassing. The results have shown that the size and morphology of the filler based on diatomaceous earth affects mechanical and rheological properties of systems based on epoxy resin. Elongation at rupture and flexural stress at rupture were both raised by up to 35%, and impact strength by up to 25%.

## 1. Introduction

The engineering of new polymer-based composites with a reduced fraction of petroleum-based material matrix is one of the trends in designing novel products containing natural raw materials. Due to their functional properties such as adhesion, flame resistance or chemical and thermal stability, epoxy resins are used in various sectors of industry. Despite having many benefits, they are fragile and have a low impact and crack propagation resistance, which considerably limits their scope of use. The curing of epoxy resins is accompanied by a very low shrink, so a cured epoxy resin precisely represents the shape and dimensions of the mold. This creates great possibilities to produce elements of different shapes [1]. However, it is still necessary to increase the toughness and strength of such a material in order to satisfy the increasing demands of specific applications [2]. This is despite the significant improvement of the already excellent resins by using them for modification and as composite matrices [3,4]. Fillers, having a high surface area/volume ratio, are generally expected to show better mechanical properties. A high surface area is usually associated with a small filler size and with extremely rough surfaces [5]. Diatoms as biogenic materials have a complex porous hierarchical structure, which makes them micrometric fillers with a highly developed specific surface. It is estimated that diatoms produce even up to 25% of organic matter in the ocean and approximately 25% of oxygen on the Earth [6]. Their cell wall is saturated with silica, which has a great potential as a raw material. The principal source of bio-silica is the so-called diatomaceous earth (diatomite). It is a mixture of diatom frustules with various sizes and morphologies, including significant amounts of damaged and broken structures being smaller than 2–3 µm.

**Figure 1:**
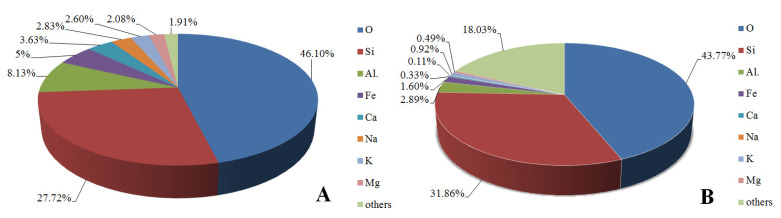
Elemental composition of Earth crust (**A**) and diatom frustule (**B**).

The Earth’s crust is composed of 93 elements, eight of which represent 99.5% of its weight. These eight most important elements are oxygen, silicone, aluminum, iron, calcium, potassium, and magnesium (Figure 1). We should point out that diatomaceous earth is, on the one hand, a representation of the most common elements existing in the Earth’s crust, and on the other hand one of the most efficient sources of biogenic silica with a great potential for commercial use.

Diatomaceous earth contains 60–95% amorphous silica. Diatoms are one of the most spectacular examples of biologically derived nano-structured materials [7]. Each diatom frustule has species-specific, regularly arrayed features: pores, ridges and protuberance [8]. The skeleton of diatoms, known as the frustule or siliceous shell, is made of nano-size silica with a three-dimensional intricate framework [9]. The mechanical properties of individual diatom frustules vary with the location of measurement, which is attributed to the different stages of the bio-mineralization process [10]. The energy required to break the frustules and the area of fracture were further shown to be relatively high, as the cracks travelled for around 40 nm through silica particles of the diatom frustules [11].

Due to their peculiar characteristics, diatoms have been recently proposed to be employed in nanotechnology as natural fillers [12]. The variety of unique frustule architectures were attractive materials for optical, mechanical, and transport properties. Diatomaceous earth has been modified in different ways and, after treatments of different kinds, used in the construction of advanced devices for light harvesting, photonics, molecular separation, sensing, and drug delivery. Diatomaceous earth has also been used in photo-catalysis, the synthesis of zeolites, removal of dyes, wastewater treatment, cement production, filtration, nanotechnology, chromatography, and mostly as an adsorbent [13].

As mentioned above, diatomaceous earth contains diatom frustules of various sizes, a lot of fractions that are mechanically damaged, as well as agglomerates and colonies. In the literature, there are methods for purifying diatomaceous earth with the use of thermal and thermo-chemical treatments [14]. Sun et al. [15] cleaned diatomaceous earth using sodium hexametaphosphate as a dispersant combined with centrifugation. Other scientists [16] used cold and hot acidic solutions. Various methods of the purification and separation of diatomaceous earth before its final use are known. Hydraulic industrial separation methods are among the most efficient. These processes use cyclones or hydrocyclones to achieve high separation efficiency. However, their main purpose is to separate sand and other inclusions [17].

We have proposed and described a method of fractioning and purifying raw diatomaceous earth which allows for the generation of a fraction with a narrow size distribution of diatom frustules.

We prepared degassed and non-degassed composites based on epoxy resin, which contained a variable amount (0, 12, 24, 36, 48, 60, 70% vol.) of base and fractionated diatomite. The prepared systems were cast in silicone molds, cross-linked and then subjected to mechanical and functional property testing and imaging with the use of a scanning electron microscope. The tests allowed us to determine whether the methodology of composite production is appropriate from the perspective of the following variables: the degassing of the prepared systems, contents of the filler (% vol.) in the tested systems, and the method of preparing the applied modifier (fractionated and base diatomite).

## 2. Experimental Section

### 2.1. Materials

**Figure 2:**
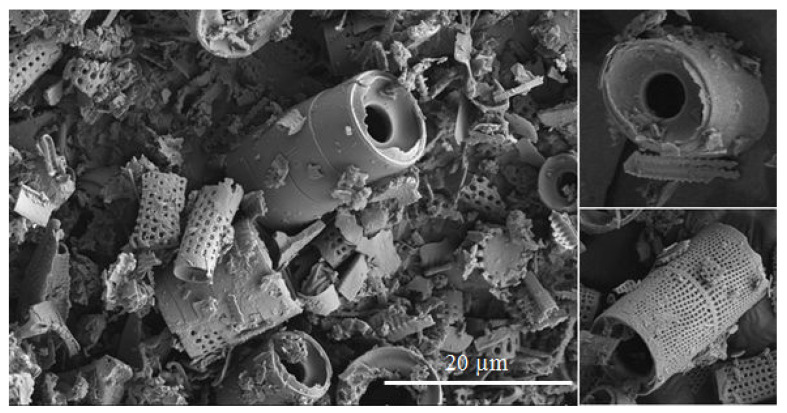
Base diatomaceous earth containing both fractured and non-fractured frustules, with a dominant content of Aulacoseira.

**Figure 3:**
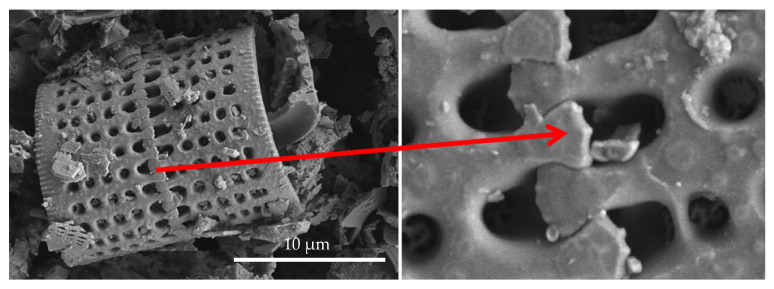
The “zipper” mechanism for creating colonies.

**Figure 4:**
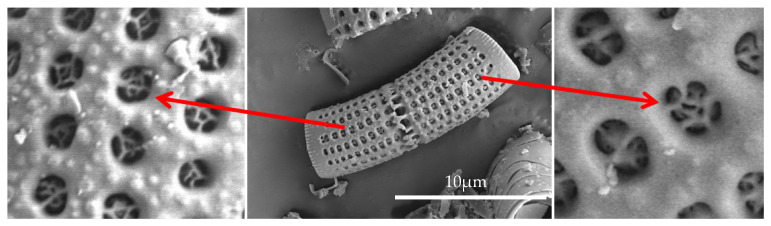
Pores present in the frustule wall.

For the tests, we used epoxy resins produced by Zakłady Chemiczne Organika Sarzyna: Epidian 601 (Nowa Sarzyna, Poland) with an epoxide equivalent of 0.50–0.55 mol/100 g and a viscosity of 700–1100 [mPa·s], cured with triethylenetetramine (curing agent Z-1). Diatomaceous earth (Perma-Guard, Bountiful, UT, USA) was derived from diatomite deposits. It was provided in a powder form and used as received for “base diatomite” preparations and fractionated according to the procedure described further for “fractionated diatomite” preparations. It mostly contains the cells of Aulacoseira, a planktonic freshwater diatom. They have a perforated scutellum specific to that species (Figure 2). Diatoms have a mechanism that allows them to create colonies (chains). The mechanism (“zipper”) is presented in Figure 3. The diversified size of diatoms is due to their life cycle. Along with cell division, diatoms shrink until they are no longer capable of dividing. They sexually multiply, as a result of which their cells reach their maximum size, which is followed by vegetative division where the cells start to shrink. For this reason, diatomaceous earth is a filler that features a high heterogeneity of particle size, which has already been mentioned (Figure 2), and a high structural heterogeneity (Figure 4) [6].

### 2.2. Sample Preparation

Raw diatomaceous earth contains broken, mechanically damaged structures which differ by the geometry of their inner structure, shape, and size. For the purpose of this study, raw diatomite was mechanically mixed with water and fractionated with the use of sedimentation. The small, broken frustule pieces were partly removed, along with bigger agglomerates/colonies, by suspending the diatomite in water and subjecting the suspension to sedimentation. Based on interacting gravitational forces, particles of different sizes settled out at different ratios.

**Figure 5:**
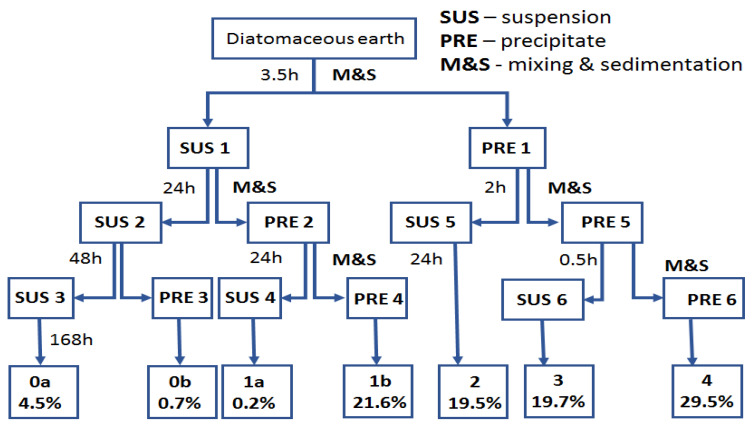
Fractionation of diatomaceous earth and the percentage share of each fraction of diatomaceous earth after fractionation.

**Figure 6:**
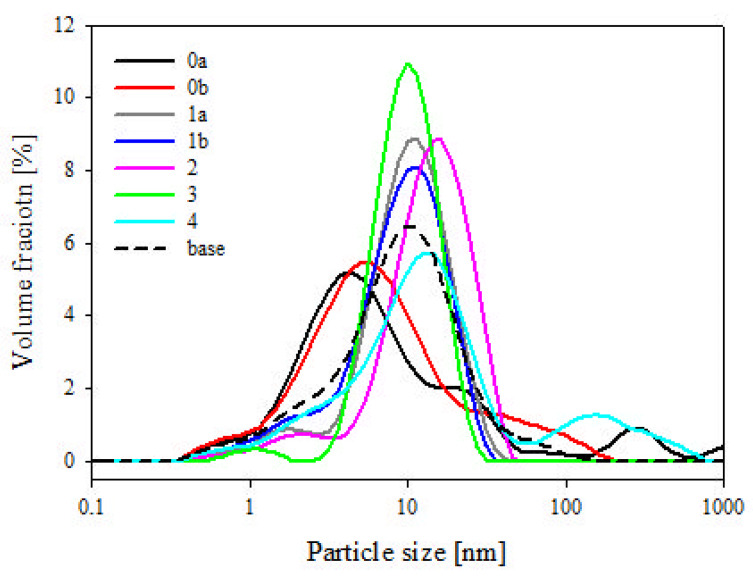
Separation of particles of fractionated diatomaceous earth by size.

**Figure 7:**
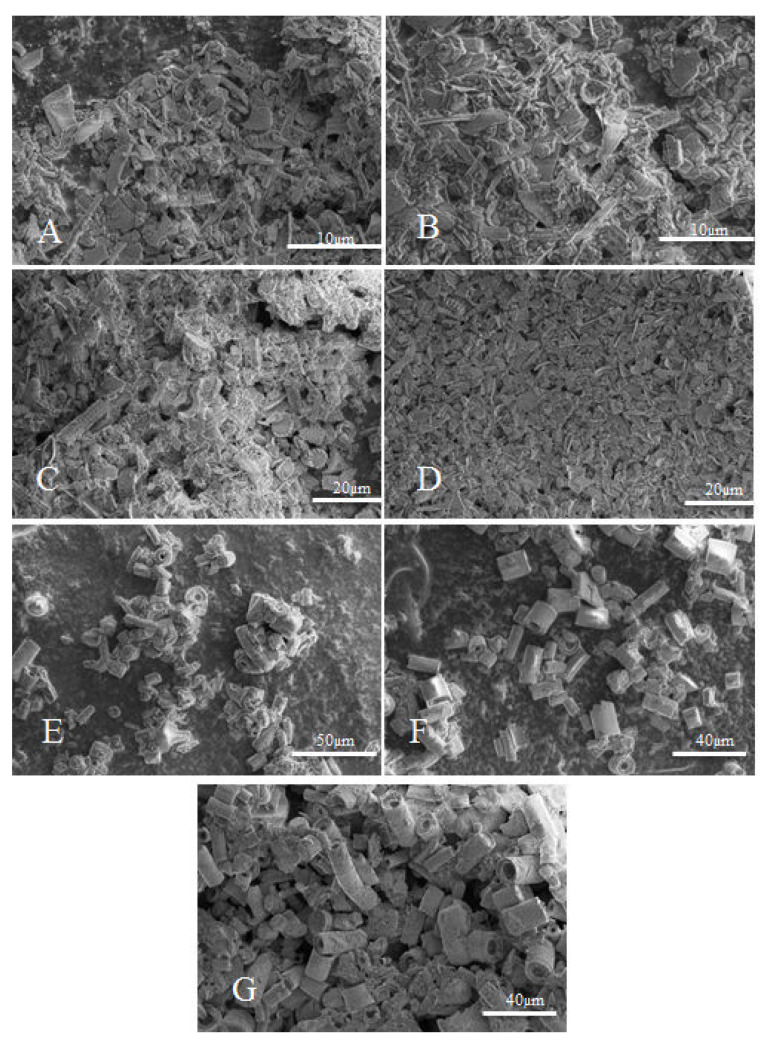
Microscopic images of fractionated diatomaceous earth (**A**) 0a, (**B**) 0b, (**C**) 1a, (**D**) 1b, (**E**) 2, (**F**) 3, (**G**) 4.

The fractionation was performed by placing 1 kg of diatomite in a 50 dm^3^ tank, adding 40 dm^3^ of demineralized water and mixing mechanically for 10 min, and leaving it for 3.5 h to cause sedimentation of the heavier precipitate fraction PRE1 (Figure 5). The next step was to pour off the suspension from above the precipitate to obtain suspension 1 (SUS1) and precipitate 1 (PRE1). SUS1 was mixed for 10 min with 40 dm^3^ of water and then left for 24 h to obtain SUS2 and PRE2. SUS2 was left for 48 h for sedimentation to obtain PRE3 (which, once dried, was marked as tail fraction 0a) and SUS3 which was left for 168 h to obtain tail fraction 0b. The PRE2 was filled with 40 dm^3^ of water, mixed for 10 min and left for 24 h to obtain SUS4 and PRE4 (which, once dried, was marked as tail fraction 1b). SUS4 was left for 24 h to obtain tail fraction 1a. The PRE1 was filled with 40 dm^3^ of water, mixed for 10 min and left for two hours to obtain SUS5, which was left for 48 h to obtain tail fraction 2. The PRE5 was filled with 40 dm^3^ of demineralized water, mixed for 10 min and left for 30 min to obtain PRE6 (fraction 4) and SUS6, which was left for 48 h to obtain fraction 3. The diatom precipitate (individual fractions) was dried with a thin layer at 80 °C to obtain a stable weight. The percentage share of each precipitate is shown in Figure 5. The total share of the fractions is 95.7%, which arises from the contents of extremely small fragments of bio-silica coming from the diatoms that did not sediment in the process and were rejected along with the operating liquid. The morphology of diatoms in each fraction was characterized by scanning electron microscopy (SEM, Hitachi, Tokyo, Japan). On the basis of dynamic light scattering (DLS) (Figure 6) and SEM analyses (Figure 7), fraction 3 was chosen for further study, as explained in the “Discussion”. The developed method allows for the purification of raw diatomaceous earth by removing smaller and broken fragments of diatom frustules and by de-agglomeration (Figure 6). Additionally, this method enables us to separate the fractions with bigger frustules (tail fraction 4) from those having a smaller geometric size (tail fraction 3); see Figure 6.

**Figure 8:**
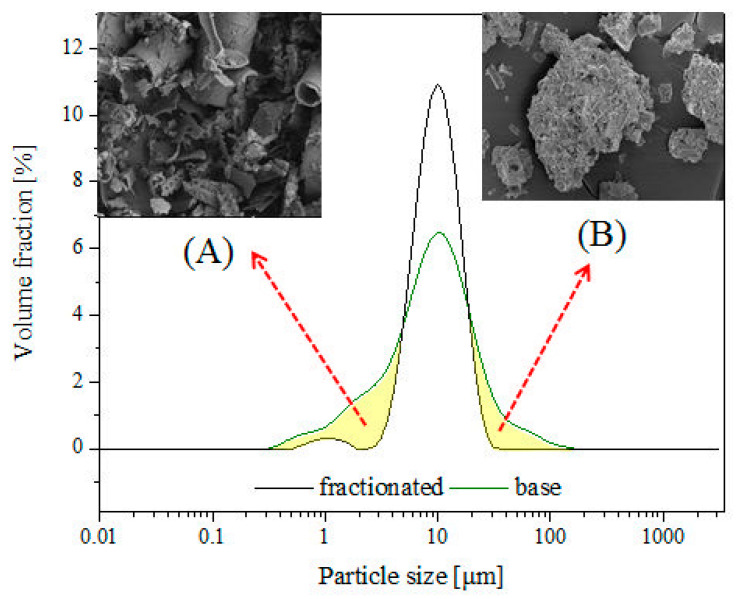
Particle size distribution for (**A**) base and (**B**) fractionated diatoms.

The studied composites of epoxy resin Epidian 601 (epoxy number (mol/100g) 0.5–0.55, viscosity at 25 °C (mPa·s) 800–1500), cross-linked with amine-based curing agent Z-1 (triethylenetetramine, viscosity at 25 °C (mPa·s) 20–30; amine number (mg KOH/g) min 1100), contained different quantities (0, 12, 24, 36, 48, 60, 70% vol.) of base and fractionated diatoms with an average particle size distribution of approx. 10 µm (Figure 8). The choice of the resin/hardener system was dictated, on the one hand, by the necessity to obtain a low viscosity of the mixture, and on the other hand, by the optimal time of its final gelation.

The epoxy resin with diatom frustules was mixed mechanically in plastic vessel, using steel propeller (600 rpm, 10 min). The samples were cast in silicone molds, as in work of Zolghadr et al. [18]. The test samples were prepared according to EN ISO 527 [19] and EN ISO 178 [20]. The tests were performed after three weeks of samples curing at 20 °C.

### 2.3. Characterization Methods

The size of the diatoms used to prepare the composites was measured with Mastersizer 3000 (Malvern Instruments Ltd., Malvern, UK). The measurements were made for the samples in water suspension (Hydro EV attachment). The parameters of measurements for wet samples were stirrer revolution speed: 2330 RPM, and ultrasound power: 70%. Fourier transform-infrared (FT-IR) spectra were recorded on a Nicolet iS50 Fourier transform spectrophotometer (Thermo Fisher Scientific, Walthan, MA, USA) equipped with a diamond ATR unit with a resolution of 0.09 cm^−1^. Spectra were collected in the 400–4000 cm^−1^ range, with 16 scans being collected for each spectrum. Contact angle analyses were performed by the sessile drop technique (5 μL) at room temperature and atmospheric pressure, with a Krüss DSA100 goniometer (KRÜSS GmbH, Hamburg, Germany). For flexural and tensile strength tests, the materials obtained were printed into type 1B dumbbell specimens in accordance with EN ISO 527-1:2012 and EN ISO 178:2010. Tests of the obtained specimens were performed on a universal testing machine INSTRON 5969 with a maximum load force of 50 kN (Instron, Norwood, MA, USA). The traverse speed for tensile strength measurements was set at 2 mm/min, and for flexural strength it was also set at 2 mm/min. A Charpy impact test (with no notch) was performed on an Instron Ceast 9050 impact machine according to ISO 179-1 [21] (Instron, Norwood, MA, USA). The density of the composites obtained was determined with the hydrostatic method using a Sartorius YCP04MS analytical balance at 20 °C with the use of distilled water (Sartorius, Göttingen, Germany). Thermogravimetry was performed using a NETZSCH 209 F1 Libra gravimetric analyzer (Netzsch Group, Selb, Germany). Samples of 5 ± 0.2 mg were cut from each composition and placed in Al_2_O_3_ crucibles. Measurements were conducted under nitrogen (flow of 20 mL/min) in the range of 30–1000 °C and a 10 °C/min heating rate. The morphology and microstructure of the prepared composites were observed by scanning electron microscopy. Samples in the form of powders and molds were dusted with cupro-nickel using a GATAN duster. Observations were conducted with a Hitachi SU 8000 scanning microscope with an accelerating voltage of 5 kV to image the preparations (Hitachi, Ltd., Chiyoda, Tokyo, Japan). The imaging was performed in three SE modes. Viscosity tests were carried out with an MCRAnton Paar 302 rotational rheometer at a shear velocity range of 0.01–100 1/s, at room temperature (Anton Paar GmbH, Graz, Austria). A 1 mm measuring gap was used, and each measurement was performed three times.

## 3. Results and Discussion

### 3.1. Particle Size Measurements by Dynamic Light Scattering (DLS)

It can be seen that the base diatomaceous earth contained a wide spectrum of particles sized from ~0.5 µm up to over 100 µm (Figure 8). According to our previous research, for the species of diatoms used for this study, the particles smaller than 6 µm can be attributed to broken frustules (pieces thereof), while particles larger than approx. 40 µm are mostly agglomerates or colonies of frustules, as well as some single specimens of a bigger size [22]. It was observed that fraction 3 obtained by sedimentation of diatomaceous earth shows much narrower distribution of particle size, ranging from approx. 3 to 30 µm, with some additional fraction of broken frustule particles and dust particles (~1 µm) (Figure 6) which, due to small size, are always present in the sedimented material together with traces of residual suspension liquid. It appears that a large portion of broken frustule particles and agglomerates of high diameter was discarded (Figure 6). The results indicate that the proposed fractionation method allows for the obtainment of a raw material with a more uniform design and morphology, which thus provides specific properties of the finished product (Figure 8).

### 3.2. Density of Composites

**Figure 9:**
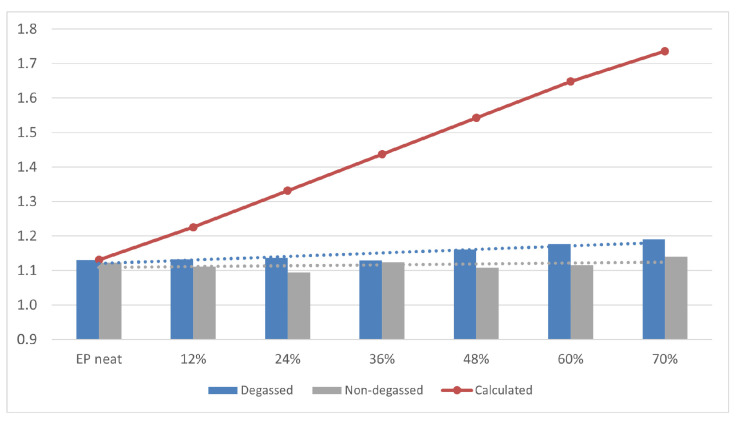
Density of degassed and non-degassed epoxy resin containing base diatomite earth.

**Figure 10:**
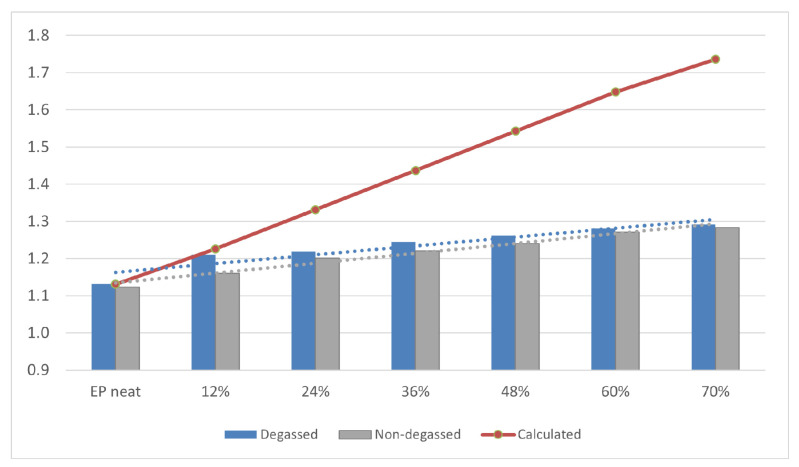
Density of degassed and non-degassed epoxy resin containing fractionated diatomite.

Density is one of the most important physical properties of the composite material. Figure 9 and Figure 10 show the determined density of composites with different contents (0, 12, 24, 36, 48, 60, 70% vol.) of base and fractionated diatoms, which have or have not been degassed. Density was determined based on the formula below:

d = V_diatom_ × d_diatom_ + (1 − V_diatom_) × d_epoxy_

V_diatom_—volume fraction of diatomite;
d_diatom_—density of solid diatomite excluding porous structures (considered 2 g/cm^3^);
d_epoxy_—density of cured epoxy (measured to be 1.12 g/cm^3^).

The bulk density of the base and fractionated diatoms is 0.271 g/cm^3^ and 0.249 g/cm^3^, respectively. For the degassed resin without diatoms, the average density was 1.131 g/cm^3^, and for the non-degassed resin, 1.080 g/cm^3^. For composites (both degassed and non-degassed) with base diatoms, we observed a slight growth in density (Figure 9). A higher density was observed for the composites containing fractionated diatoms, both degassed and non-degassed (Figure 10). A system containing 70% vol. of diatomaceous earth has a density of nearly 1.3 g/cm^3^, which is almost 10% higher than in a system containing the same amount of base diatoms.

**Figure 11:**
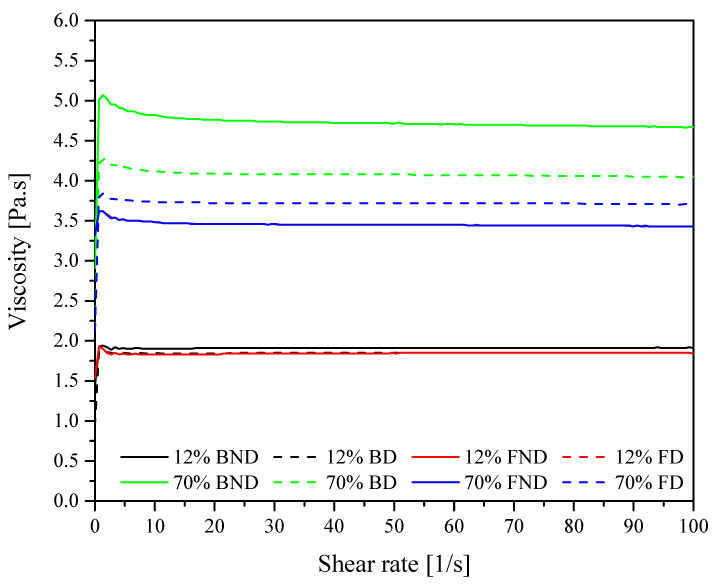
DMA rheology diatoms/epoxy resin composition, BND—base diatomite non degassed, BD—base degassed, FND—fracionated non degassed, FD—fracionated degassed.

An increase in density up to 1.3 g/cm^3^ was also observed by [5], who used centric type diatom frustules as filler in an epoxy resin (degassing was applied) with a weight percentage of 15%. It has been observed that an increasing number of supplied fractionated diatoms went along with a linear growth in the density of composites obtained. Deviation from the theoretical value calculated for fully degassed composites with frustules completely filled with epoxy (Figure 9 and Figure 10) arises from the fact that resin does not fully penetrate into the diatom structure and the nanostructure of the diatom frustule. We can also observe silica substructures present in frustule holes (Figure 4). Due to viscosity, despite the applied degassing process, the resin is not capable to penetrate into diatom frustules, since the holes are covered by the substructures. Diatomaceous earth is characterized by quite a low specific weight due to its highly porous structure, being much lower than that of neat epoxy resin, but its main component is nano- and microstructured silica, which itself is characterized by a density of ~2 g/cm^3^. The frustules’ density is defined as 1.4–2.2 g/cm^3^ [23]. Therefore, along with a successful infusion of frustules with the epoxy resin, a raise in the composites’ density is to be expected, along with increasing loading; however, it is highly dependable on the fraction of air eliminated from the frustules during infusion. Additionally, mostly for base diatomite earth, with increasing loading, the effect of composition degassing on the material density becomes more substantial, as the diatomite traps significant amounts of air. Therefore, a bigger difference between degassed and non-degassed samples can be observed. Moreover, in general, higher values of density were observed for the samples with fractionated diatomite. This effect can be linked to stronger the thixotropic effect of micron-sized particles of broken frustules, as proven by (DMA) rheology (Figure 11), which in turn causes the entrapment of more air in the samples and bigger differences between degassed and non-degassed samples for base diatomite earth. It can be seen that with low loadings, viscosity is low and barely dependable on the diatomite type or degassing procedure. With increasing loading, viscosity raises significantly and the differences between diatomite types become substantial, as base filler imparts much higher viscosity. A reduction in the viscosity of base diatomite earth-loaded epoxy after degassing may be due to the agglomeration of micron-sized particles during degassing.

### 3.3. FTIR Analysis

**Figure 12:**
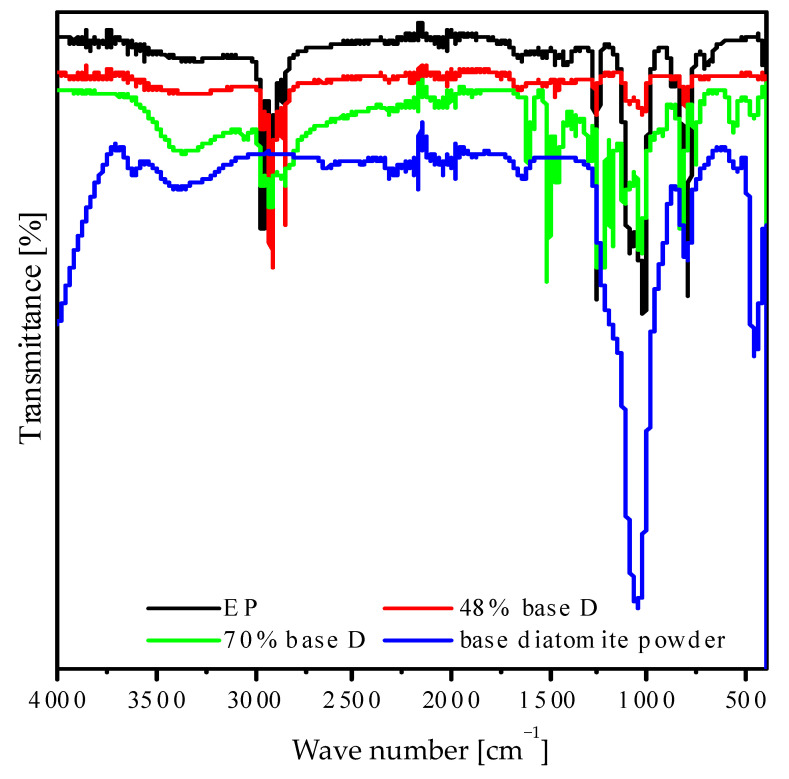
FT-IR spectra of degassed composites containing base diatoms and base diatomite powder. Base D: base diatomite. EP: neat epoxy resin.

Figure 12 shows the FT-IR spectra of degassed composites containing base diatoms. For resin samples, we can observe characteristic bands at a wavelength of 2950–2800 cm^−1^ for C–H aliphatic bonds, 1450–1400 cm^−1^ for C–O bonds, 1258 cm^−1^, and 1100–650 cm^−1^ for different C–H bonds [24]. The strong band at 2962 cm^−1^ is typical for the vibration of CH, CH_2_, and terminal methyl groups. The peak at around 864 cm^−1^ can be assigned to 1,4-substitution of aromatic ring for epoxy resin. The band characteristics of ester groups, representing the C–O–C bond stretching, are found at 1258 cm^−1^ and 1015 cm^−1^ [25]. For degassed composites with base diatoms, there emerged weak bands at wavelengths of 3566 and 3304 cm^−1^, related to stretching vibrations of O–H bonds in silanol groups as well as in hydrogen-bonded molecular water [26]. We also observed an additional band at a wavelength of 797 cm^−1^, which is caused by symmetric bending vibrations of Si–O–Si bonds [27]. The band at 1646 cm^−1^ is due to bending vibrations of H–O–H bonds of bound molecular water [22]. We additionally observed bands at a wavelength of 1015 cm^−1^, which could be attributed to the asymmetric stretching of Si–O–Si bond, while the 661 cm^−1^ belong to symmetric stretching [28].

### 3.4. Mechanical Properies

As for the mechanical properties of the obtained composites, we examined their tensile strength, three-point flexural strength, and impact strength.

#### 3.4.1. Tensile Strength

**Figure 13:**
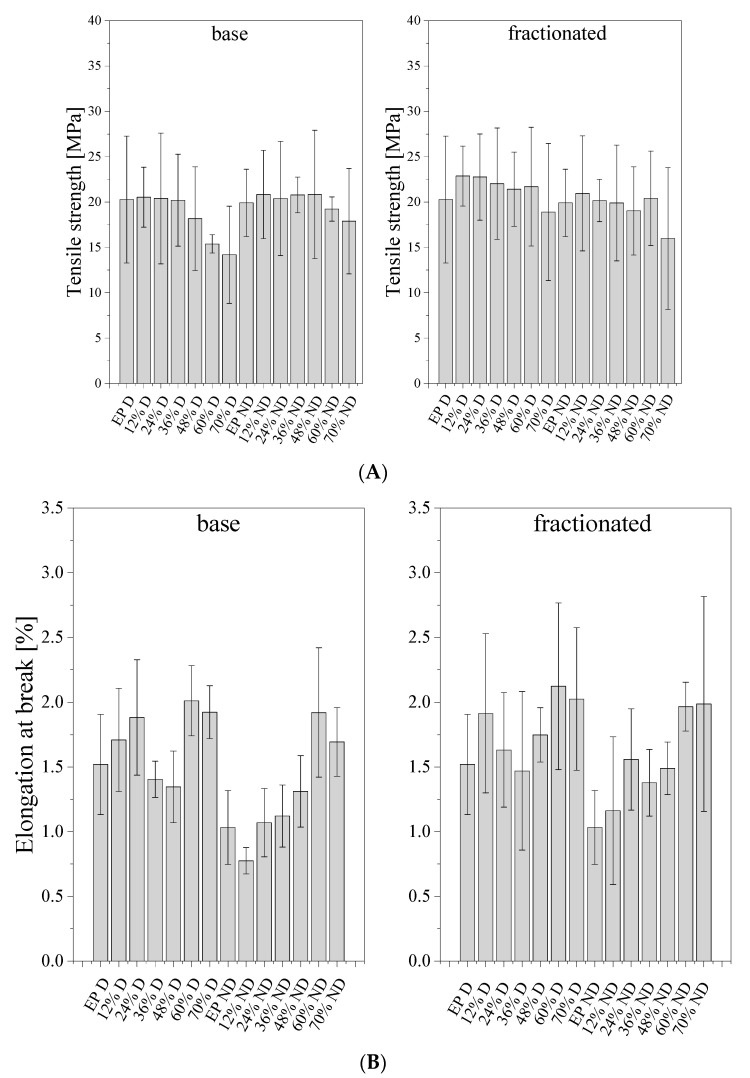
Tensile strength (**A**) and elongation at break (**B**) for degassed (“D”) and non-degassed (“ND”) composites containing base diatoms and fractionated diatoms.

Figure 13 shows the results for the tensile strength (A) and elongation at break (B) of the prepared composites. The degassed samples of neat epoxy were characterized by a higher tensile strength than the non-degassed counterparts, that is 19.91 and 18.26 MPa, respectively. For base diatomite, at low filler concentration, a slight increase in tensile strength was observed. At higher loadings, a small decline was observed, down to 15.09 and 14.82 MPa for 70% degassed and non-degassed compositions, respectively. For the compositions with fractionated diatomite, the tensile strength reached higher values than for the composites with base diatomite, up to 22.78 MPa for 24% composition. As we know, the strengthening effect of the filler is determined by interfacial action forces, which largely depend on the break-up of the filler and the development of the surface. It proves that the elimination of agglomerated particles during diatomite preparation, as well as the degassing of the epoxy composition both have positive effects on the mechanical properties of the composites obtained. Tensile strength may be linked to the amount of resin introduced into the frustules’ internal structure during composition degassing, as observed in SEM images (see Microstructural analysis). Frustules with more resin introduced into their porous structure and penetrating inside their internal volume induce slightly more mechanical reinforcement, due to improved mechanical contact between the matrix and the filler. In the work [8] that studied calcined and natural frustules filled epoxy matrices, they observed variations of tensile elastic modulus and tensile strength with vol. % of frustules addition. In the work, two main tensile failure mechanisms were described for calcinated frustule filled epoxy composites: frustules debonding and frustules fracture/crushing, followed by partial pull-out. Similar failure mechanisms were observed with SEM imaging during this investigation. Debonding and pull-out may be the reason for small levels of difference in mechanical properties when comparing neat epoxy resin and differently filled composites thereof—the limited wetting of the filler surface by the matrix epoxy.

The elongation at break was also investigated (Figure 13B). Due to the brittleness of epoxy resins being one of their main weak spots, as well as the sample preparation method, it was difficult to observe a particular trend for the elongation at break, as the results show a rather high standard deviation. The non-degassed resin without diatoms showed lower elongation at break (1.033%) than the degassed resin (1.520%). The samples containing base frustules without degassing showed slightly lower elongation at break than their counterparts with fractionated filler. It can be explained on the basis of the frustule agglomerates observed in the base diatomite earth forming defect sites. Additionally, for low loadings, the entrapped air in both systems also contributes to material failure at low elongation. Likely for this reason, the difference was poorly visible for degassed samples. In general, as expected, the degassed samples showed improved elongation at break when compared to the non-degassed ones. From the practical point of view, for the studied systems, it is important that even at high loadings the obtained composites show mechanical parameters comparable to those of the neat epoxy resin, being a reasonable option for more eco-friendly materials with a high content of biogenic filler.

#### 3.4.2. Flexural Strength

**Figure 14:**
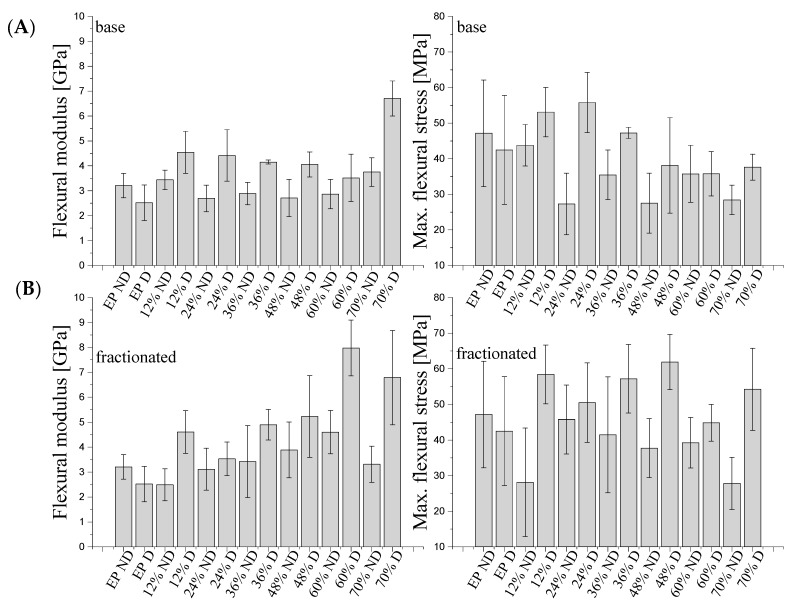
Flexural modulus and max. bending stress for degassed and non-degassed (“D” and “ND”) composites containing base (**A**) and fractionated (**B**) samples diatoms.

Figure 14 shows the maximum flexural stress and flexural modulus for degassed and non-degassed composites containing base (A) diatoms and fractionated (B) diatoms. The flexural stress for degassed and non-degassed epoxy resin was 42.48 MPa and 47.15 MPa, respectively. For degassed samples, the stress at three-point bending gradually increases at base diatom contents of 12 and 24% vol. to reach 53.09 MPa and 55.78 MPa, respectively, and then, at base diatom contents of 48, 60 and 70% vol., the stress gradually decreases to reach values down to 35.79 MPa. For degassed composites containing fractionated diatoms, the flexural stress reaches higher values than for the composites with base diatoms, the maximum value of 61.87 MPa being reached for 48% vol. The non-degassed samples showed lower values of flexural stress than the degassed ones, both for base and fractionated diatoms. For the non-degassed samples, we observed much lower values regardless of the filler used. In general, the conclusions are similar to those for tensile tests and prove that both the purification of diatomite to the desired fraction containing single, undamaged frustules, and the degassing of the composition give the best results and improve flexural strength of the composites at the highest loadings, while leaving the flexural modulus similar to the base value. Additionally, for non-degassed composites, a decline of the flexural modulus was observed, as the air gaps increase the elasticity of the material.

For degassed composites, the maximum value of flexural modulus is higher than for degassed composites. The initial value for resin with degassing was approx. 2.5 GPa. Degassed composites with fractionated diatoms showed a higher maximum flexural modulus than those with base diatoms. This could be due to the uniformity of the raw material used. However, in both cases (base and fractionated diatoms), at 70% vol., the value of flexural modulus reached around 6.7 GPa. For non-degassed resin without a modifier, the value of flexural modulus was 3.2 GPa. The addition of 12 and 70% vol. of base diatoms caused a slight growth of the maximum flexural modulus up to 3.7 GPa. In other cases, the value was even higher than the initial value. The use of fractionated diatoms also did not cause any considerable increase in flexural strength. The addition of 36–60% vol. of fractionated diatoms caused a slight increase in the value, and the further addition of the modifier resulted in a significant deterioration of properties. The maximum value of flexural stress, existing at a 60% content of fractionated diatomite, is approximately 4.6 GPa.

The use of fractionated diatoms (degassed samples) in a polymer matrix caused a growth in flexural stress. The non-degassed samples showed lower flexural stress values than the degassed samples, both for base and fractionated diatoms, as the presence of air bubbles reduced the strength of the material. Due to degassing, we could see more clearly the strengthening impact of the filler. For the determination of the flexural modulus, the degassing of the sample was important. Non-degassed samples showed comparable (and in certain cases, reduced) flexural modulus values, which were caused by a high share of air and a weaker resin–filler interaction. Changes in flexural strength were observed only for degassed composites, where we observed a slight increase in the flexural modulus. The presence of air bubbles had a considerable impact on the results.

#### 3.4.3. Impact Strength

**Figure 15:**
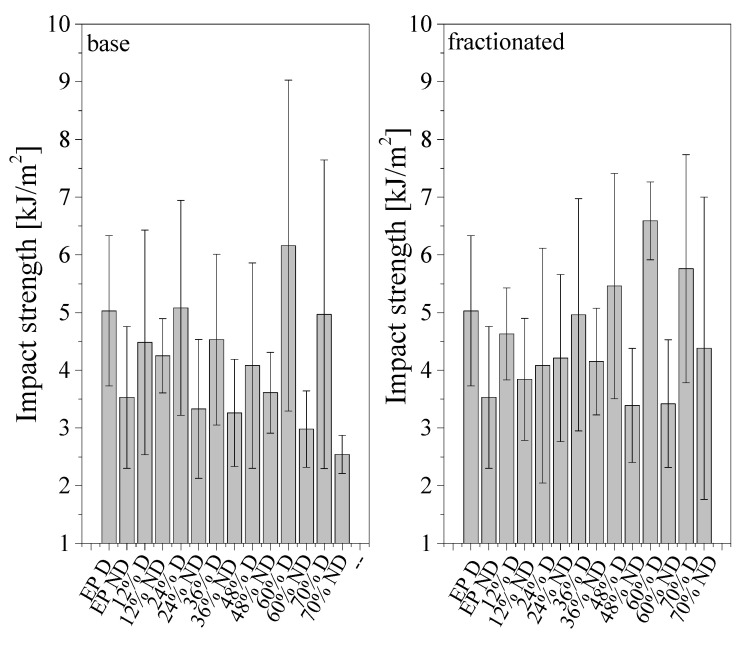
Impact strength for degassed and non-degassed composites containing base and fractionated diatoms.

For impact strength (Figure 15), two main reasons for decreased values of non-degassed composites should be considered. One of them is the presence of air bubbles introducing mechanical discontinuities, and the other one may be the lower bonding between the matrix and the filler, which in turn causes tearing of the frustules from the resin and increased crack propagation during impact. Impact strength is usually lower for non-degassed samples; this is caused by the presence of air bubbles, which have a significant impact on mechanical properties. A higher impact strength is shown by the samples modified with fractionated diatomite, which may be caused by a more even distribution of diatom particles. Most of the analyzed samples have an impact strength ranging from 2 to 5 kJ/m^2^. The values grow along with increasing filler content. The best results were attained by degassed samples containing 60% vol. of diatomite (base and fractionated: 6 and 9 kJ/m^2^, respectively). In general, the impact strength was comparable to the base material regardless of the filler loading, and only sample degassing played a visible role in increasing the material durability.

In the measurements, we observed a large dispersion of results. It may be due to the following reasons: (a) the structural heterogeneity of the composite; (b) the use of silicone molds for casting, whose edges leave defects in the composite (as confirmed by SEM, the edges of the samples were highly irregular); (c) the presence of air bubbles, marking the start of cracking (the presence of air bubbles (both for degassed and non-degassed composites) at random spots of the samples); (d) cracking of the agglomerates for base diatomite. Therefore, in further tests, we may consider whether it is appropriate to use a silicone mold to prepare epoxy resin composites.

### 3.5. Water Contact Angle

**Figure 16:**
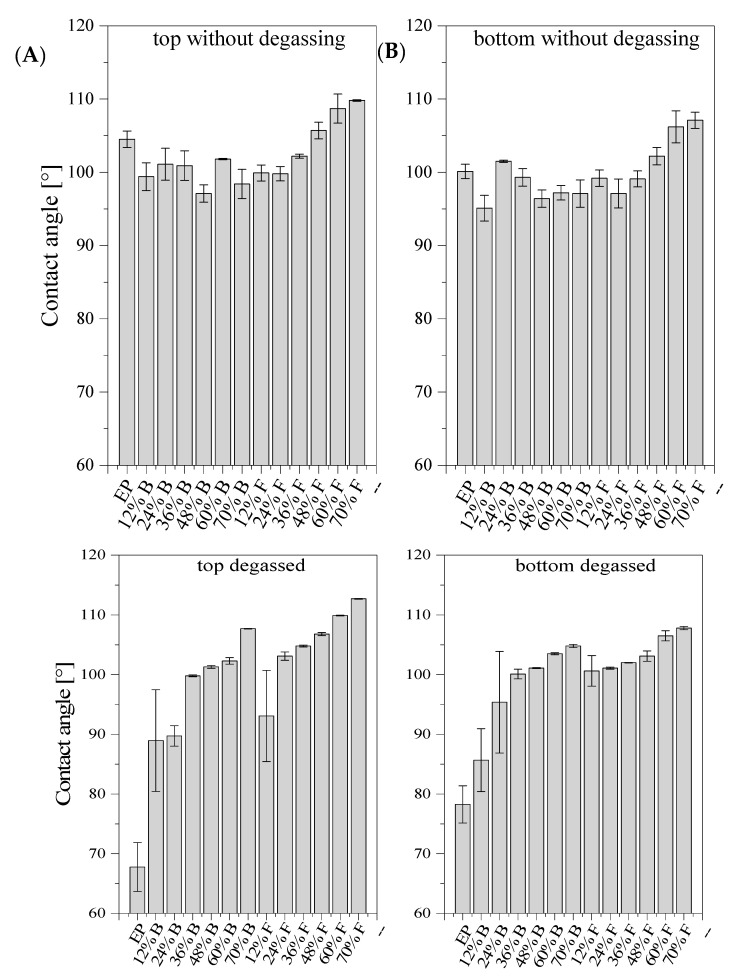
Contact angle bottom (**A**) and top (**B**) for degassed and non-degassed composites containing base (“B”) diatoms and fractionated (“F”) diatoms.

**Figure 17:**
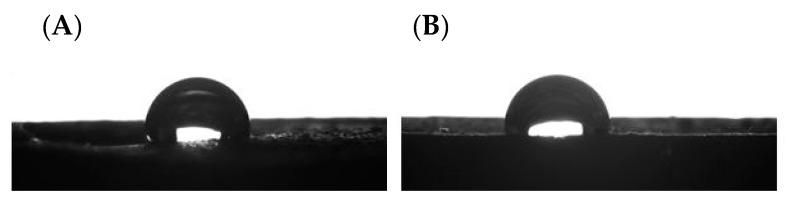
Droplets applied on the bottom part of the dumbbells for samples containing 70% vol. (**A**) with non-degassed base diatoms, and (**B**) with non-degassed fractionated diatoms.

Wettability is a physical feature of materials that specifies their interaction with liquids, mostly water, and determines the basic material properties such as adhesion. The contact angle is an important parameter for determining the hydrophobic or hydrophilic nature of polymers. The contact angle test was conducted on samples shaped as dumbbells. We tested the top and bottom parts of the dumbbells. The angle value for each type of composite was given as the average value obtained in the measurements (Figure 16). Example pictures of a droplet are presented in Figure 17. The samples of degassed and non-degassed resin without diatoms showed hydrophilic properties. As we know, 85% of diatomite consists of SiO_2_. The stable tetrahedral structure of SiO_2_ makes diatomite a hydrophobic material [29]. However, depending on the degree of purification, diatomite is available with hydrophilic or hydrophobic surface properties [30]. Composites modified with diatomaceous earth showed hydrophobic properties, both in the top and bottom parts. This was except for systems with degassing that contained 12 and 24% vol. of base diatoms and 12% of fractionated diatoms. The values of contact angle in this case are below 90°. Hydrophobic properties of the tested systems increased along with the growing concentration of modifier in the samples and showed higher values for the systems containing fractionated diatomite. We may therefore conclude that the incorporation of diatomite into the epoxy resin matrix resulted in a considerable increase in the overall hydrophobicity.

### 3.6. Changes in Weight/Rate of Changes with Increasing Temperature (TG/DTG) Analysis

**Figure 18:**
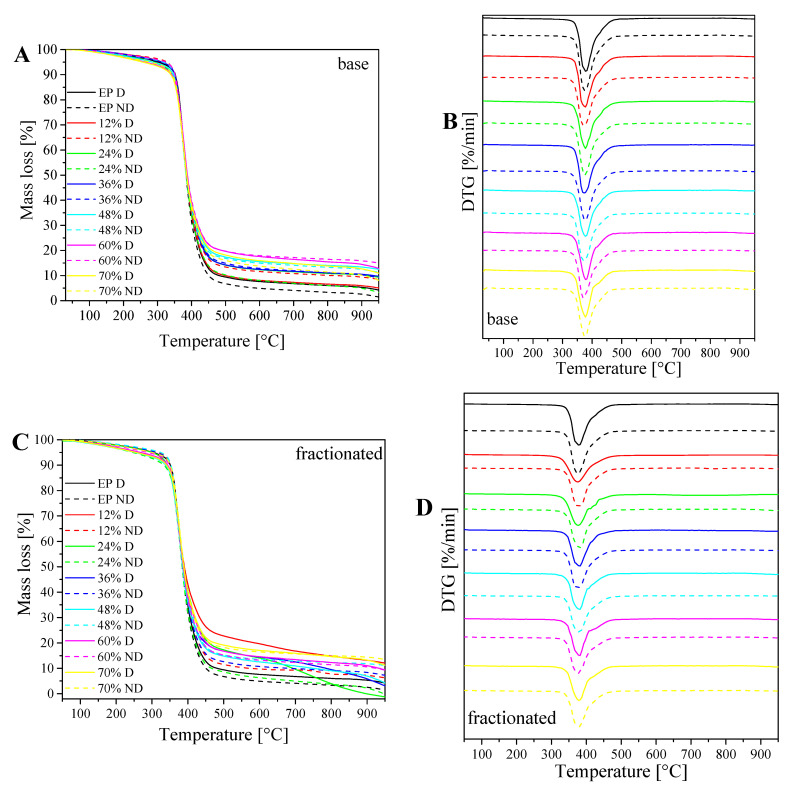
Changes in weight (TG) and the rate of changes with increasing temperature (DTG) curves for degassed and non-degassed composites containing base diatoms (**A**,**B**) and fractionated diatoms (**C**,**D**).

TG thermograms and DTG for degassed and non-degassed composites containing base (A,B) and fractionated diatomite (C,D) (Figure 18). In the samples obtained, we measured the changes in weight (TG) and the rate of these changes along with increasing temperature (DTG). The decomposition of both fractions of diatomaceous earth in the atmosphere takes place in a single stage. This is due to the decomposition of the epoxy resin itself. The addition of diatomite, regardless of the fraction used, increases thermal stability of resin–filler systems. Temperature at a 5% weight loss is, in each case, lower for degassed samples than for non-degassed samples, by nearly 10% maximum (for 48% fractionated samples), whereas the samples containing fractionated diatoms generally show lower temperature values at a 5% weight loss than base diatoms. Temperature at the maximum weight change rate is at a similar level for all the analyzed systems, and ranges from 375 to 381 °C.

### 3.7. Microstructural Analysis

**Figure 19:**
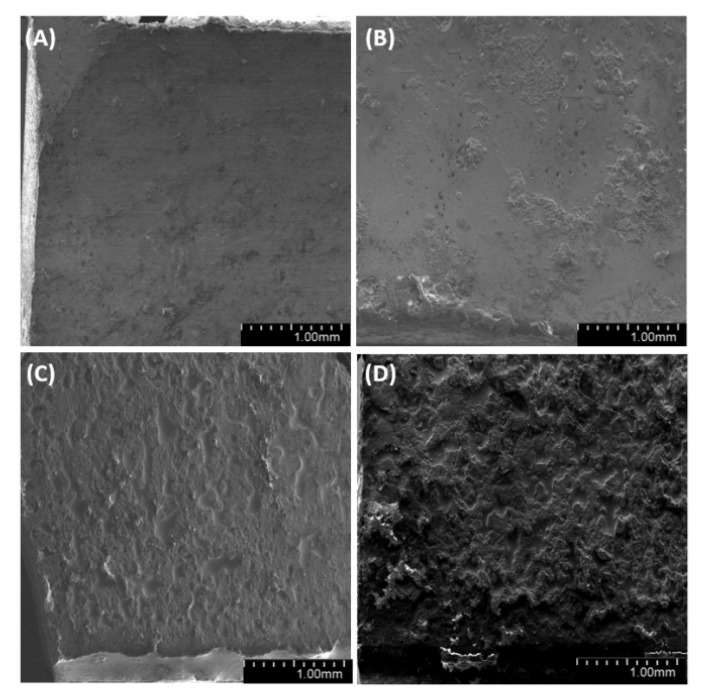
SEM images of the surface of samples with 36% vol. of diatoms (**A**)—base deg, (**B**)—base no deg, (**C**)—fractionated deg, (**D**)—fractionated no deg.

**Figure 20:**
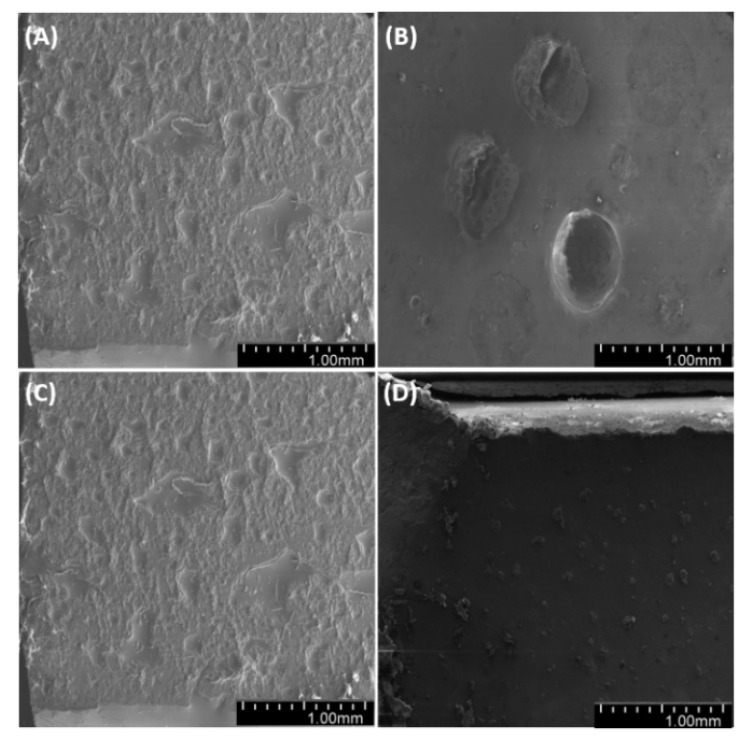
SEM images of the surface of samples with 48% vol. of diatoms (**A**)—base deg, (**B**)—base no deg, (**C**)—fractionated deg, (**D**)—fractionated no deg.

**Figure 21:**
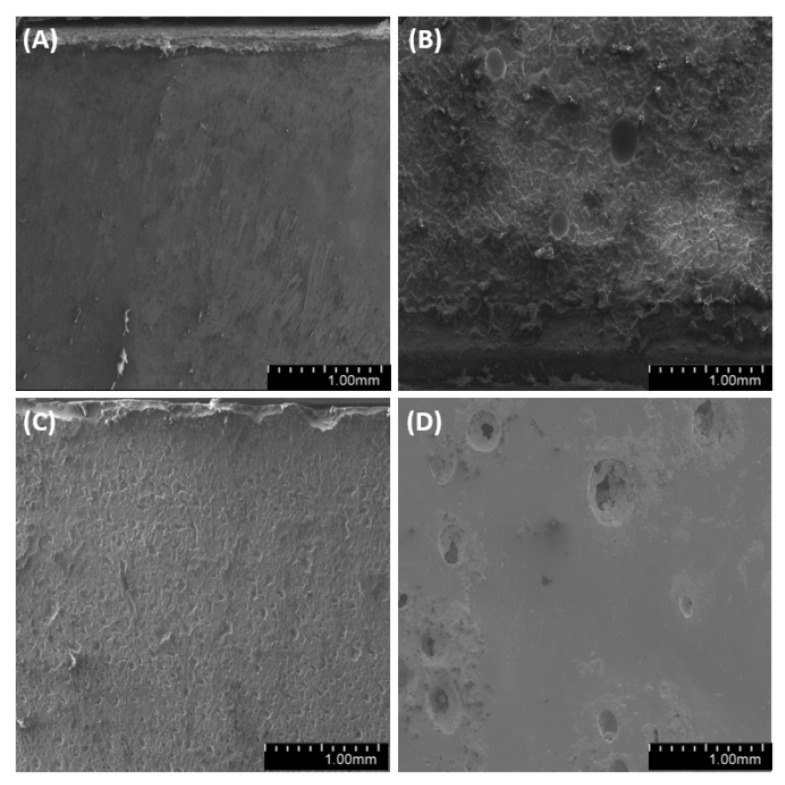
SEM images of the surface of samples with 70% vol. of diatoms (**A**)—base deg, (**B**)—base no deg, (**C**)—fractionated deg, (**D**)—fractionated no deg.

We have analyzed the side, fracture and meniscus of the samples. For both degassed and non-degassed samples, we observed air bubbles, which are smaller for degassed samples. The observations of sample surfaces (Figure 19, Figure 20 and Figure 21) allowed us to conclude that the surface is rough. The superficial irregularity decreased along with the growing concentration of diatoms in the system. On the surface of non-degassed samples, we can clearly see air bubbles, while in the degassed systems the air bubbles are either small or do not exist at all.

**Figure 22:**
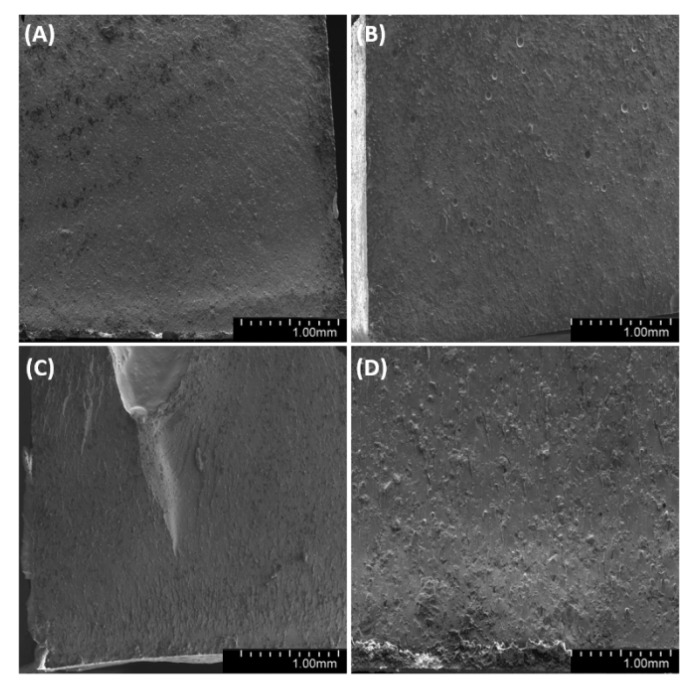
SEM images of the fractures of samples with 36% vol. of diatoms: (**A**)—base deg, (**B**)—base no deg, (**C**)—fractionated deg, (**D**)—fractionated no deg.

**Figure 23:**
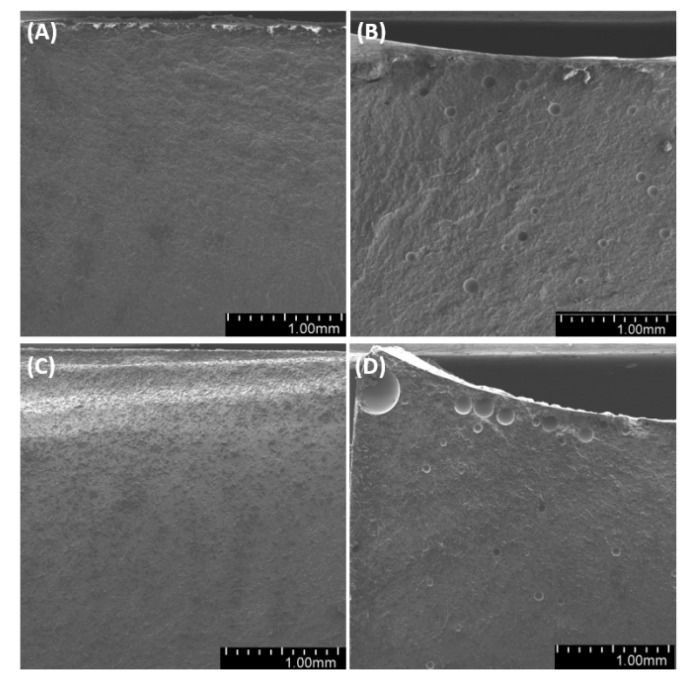
SEM images of sample fractures: 48% vol. of diatoms: (**A**)—base deg, (**B**)—base no deg, (**C**)—fractionated deg, (**D**)—fractionated no deg.

**Figure 24:**
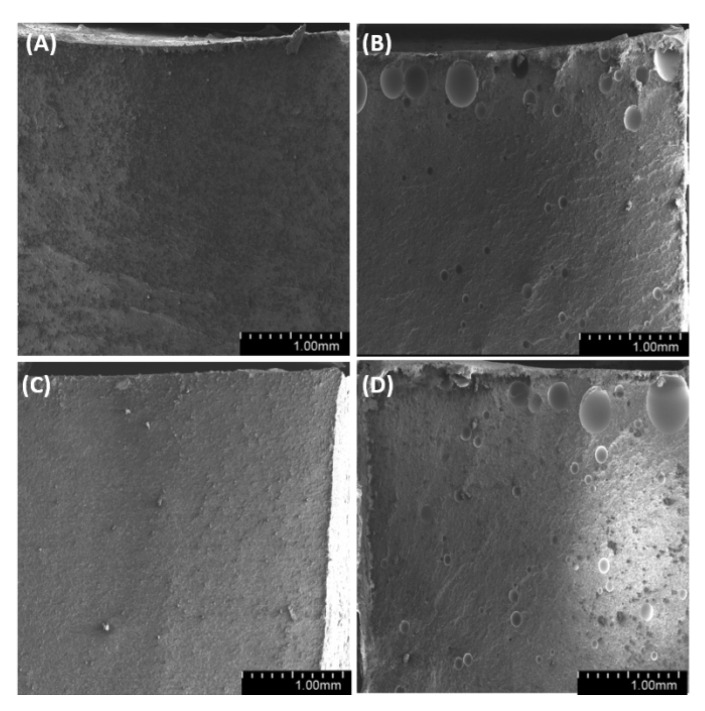
SEM images of the fractures of samples with 70% vol. of diatoms: (**A**)—base deg, (**B**)—base no deg, (**C**)—fractionated deg, (**D**)—fractionated no deg.

Observations of sample fractures (Figure 22, Figure 23 and Figure 24) have shown that the analyzed surface is mostly homogeneous. In non-degassed samples, we can observe small air bubbles located on the entire fractured surface as well as larger bubbles located in the upper section of the sample (closer to the meniscus). The fractured surface of degassed samples shows a similar degree of uniformity regardless of diatomite concentration. The samples containing fractionated diatoms feature air bubbles that are slightly bigger than those in the samples containing base diatomite.

**Figure 25:**
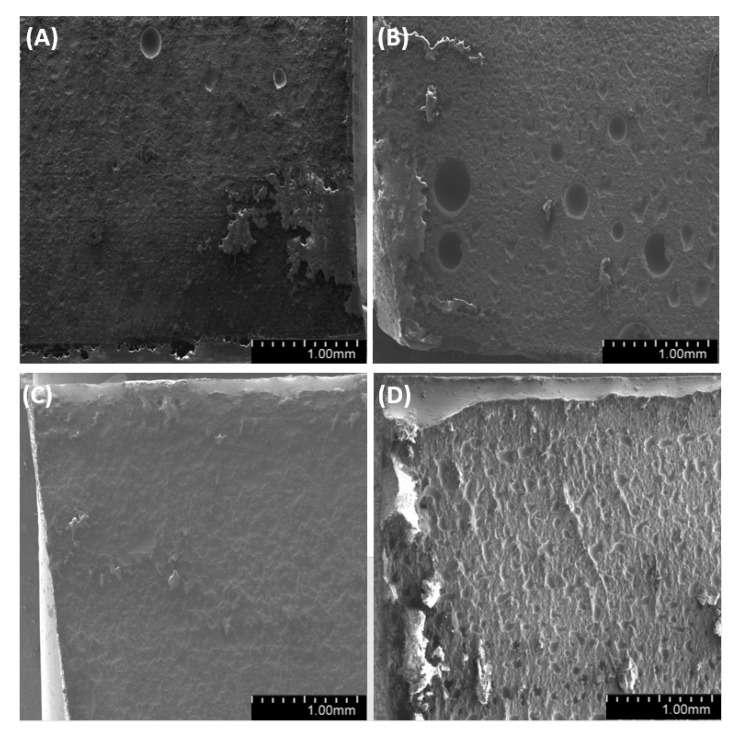
SEM images of mold wall with 36% vol. of diatoms: (**A**)—base deg, (**B**)—base no deg, (**C**)—fractionated deg, (**D**)—fractionated no deg.

**Figure 26:**
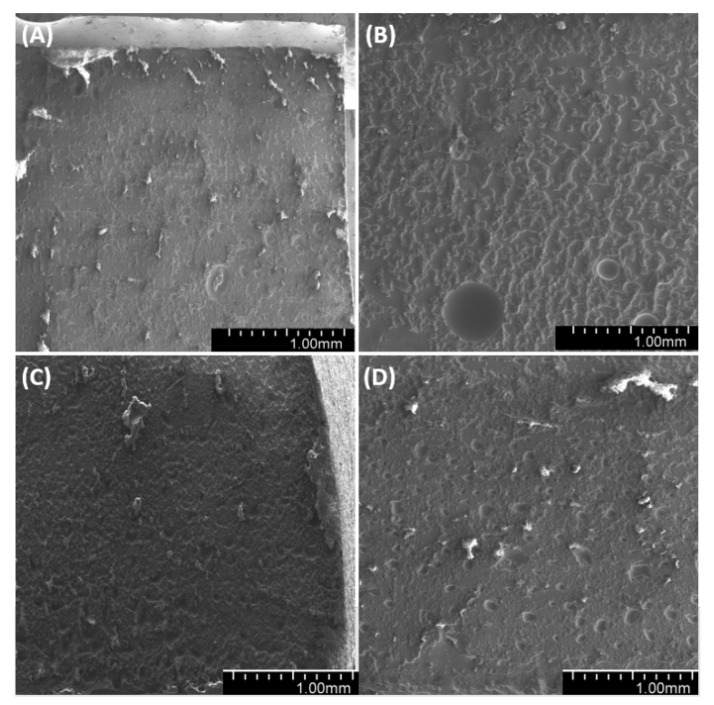
SEM images of mold wall with 48% vol. of diatoms: (**A**)—base deg, (**B**)—base no deg, (**C**)—fractionated deg, (**D**)—fractionated no deg.

**Figure 27:**
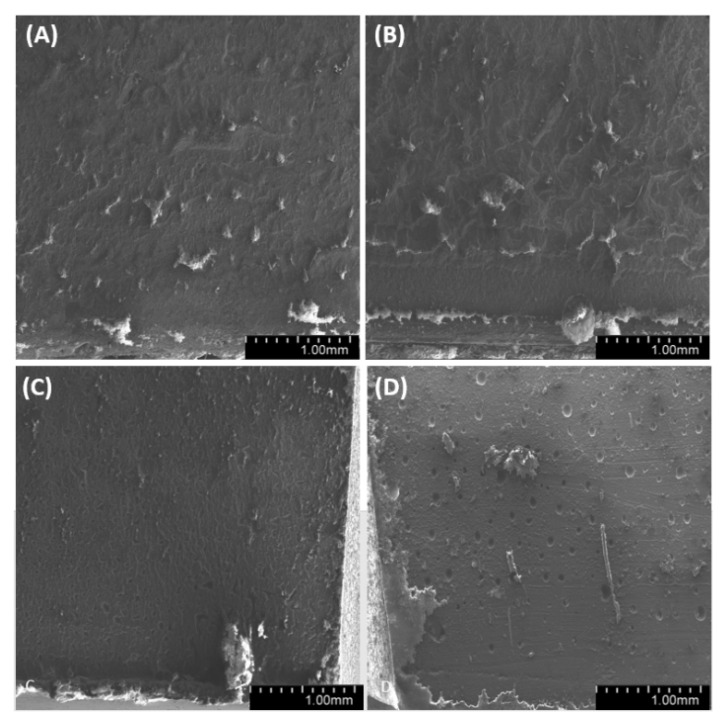
SEM images of mold wall with 70% vol. of diatoms: (**A**)—base deg, (**B**)—base no deg, (**C**)—fractionated deg, (**D**)—fractionated no deg.

**Figure 28:**
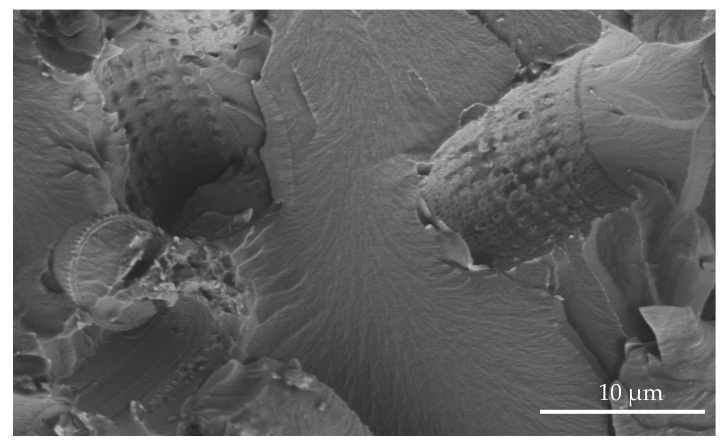
SEM image of diatoms filled with resin (**right**) and the mark after diatom debonding (**left**).

By observing the wall of the mold that contained the tested samples (Figure 25, Figure 26 and Figure 27), we found a significant heterogeneity in its structure, which, in the results, was also present in the composite samples. In most of the examined systems, the mold walls have irregular projections which are due to the fact that the samples adhere to the silicone molds. As the diatomite concentration in the system raises, the surface roughness drops. Systems that contain over 48% vol. of diatomaceous earth showed a lower inclination to adhere to the silicone mold during sample preparation, so the mold walls in those systems seems to be smoother. Non-degassed systems show the presence of air bubbles. Their size depends both on diatom concentration in the system and on filler preparation. Samples with base diatoms without degassing have bigger air bubbles, while the samples with fractionated diatoms have air bubbles that are much smaller. Along with the growing quantity of diatoms in polymer matrix, the share of bubbles is usually lower. In addition, in all the tested samples, whether degassed or non-degassed, we observed that diatoms were torn off the polymer matrix, leaving a clear mark, or were sheared and partially left in the matrix. This proves that the place where a diatom frustule is bonded with resin is more exposed to damage than the structure of base resin. This arises from low adhesion between the mineral and organic parts of the composite. Additionally, as mentioned in Results—Mechanical Properties, in all the tested samples we observed the supersaturation of resin into the diatomic structure (Figure 28 on the right) as well as the mark it left after debonding (Figure 28 on the left). We have noticed that the resin partially penetrated into the micropores in diatom frustule, creating mechanical links with the polymer matrix.

## 4. Conclusions

The tests were aimed to experimentally verify the assumptions on the technology of the fractionation of raw diatomaceous earth to obtain resin–diatomite composites for a specific fraction of diatom frustule structures. We have verified the impact of fractionation as a function of diatom content as well as of process factors such as the degassing of samples. The applied methods of separating diatoms by fractionation and sedimentation enables the removal of diatom agglomerates and a large part of fractured frustules and dusts. It is possible to separate diatom frustule fractions with a specific size distribution. The effective density of diatoms is higher than the density of resin, so the density of obtained composites grows along with the increasing weight share of diatoms. What is important, however, is that the relation between density and weight share of diatoms is not linear and depends on the size of diatom frustule and on the degassing degree of the mixtures. Non-fractionated diatoms increase resin viscosity, which hinders its effective degassing. Samples obtained by degassing technology and those with fractionated diatoms featured higher density, which proves that the tested composites can be considered as a system of three phases: diatoms + resin + pores filled with gas. Based on calculations, we have estimated the expected density of composites with a theoretical 100% filling level, which has shown that with the applied method of preparing composites, diatoms are not completely filled with resin. At the same time, based on SEM, we can conclude that this is not required to obtain good wetting of the filler surface with resin, plus it allows us to obtain lighter materials.

The degassed composites (so those with fewer pores) featured higher tensile strength. Their mechanical properties were also affected by the method of diatom preparation. Samples with fractionated diatoms showed higher tensile strength values than composites with base diatoms, but the effect was more noticeable for non-degassed systems. The slight differences in the strength of samples containing different amounts of filler could be caused by the separation of diatoms from resin during tension (debonding, pulling out). This was similar for elongation at break, which was higher for composites with fractionated diatoms, especially for non-degassed systems. As we know, the strengthening effect of the filler is determined by interfacial action forces, which largely depend on the break-up of the filler. Since diatoms after fractionation are smaller than diatoms as delivered (they do not contain the agglomerates imaged by SEM), the composites based on them feature higher tensile strength. An important observation is that the composites maintain good strength parameters despite a high degree of filling (even for 70% vol.). The addition of fractionated diatoms to the matrix in sample degassing technology affects the hydrophobicity of their surface. The effect is due to the formation of surface microstructure in a diatom frustule–epoxide resin arrangement. The addition of diatoms (whether base or fractioned) caused an increase in the thermal stability of resin–filler systems. The comparative tests carried out (without degassing and degassing) allowed us to conclude that the presence of gas in the composite does not result from the degassing method (which could be a methodological objection in the process of evaluating our results) but is related to the release of gas from the inside of the diatom during the cross-linking process (system heating up). Studies have shown that standard methods adopted as good industrial practice are not sufficient to remove the air contained in diatom shells. The specificity of diatom frustules’ morphology is unusual for commonly used fillers. They have a hierarchical porous structure (nanopores, micropores and macro pores). It is necessary to use other factors, such as auxiliary means, but this is not the subject of this work.

Tests have shown that diatom frustules can be used as a biologically friendly filling phase of natural origin in polymer composites, which allows for the reduction in the consumption of production with a high degree of chemical treatment such as epoxy resins.

## Data Availability

Data available in a publicly accessible repository.
